# Fear and Impact of COVID-19 Among Post-Infected Adults: Types and Associations with Quality of Life and Post-Traumatic Stress Symptoms

**DOI:** 10.1007/s44197-024-00333-2

**Published:** 2024-12-02

**Authors:** Tinh X. Do, Ha-Linh Quach, Thi Ngoc Anh Hoang, Thao T. P. Nguyen, Lan T. H. Le, Tan T. Nguyen, Binh N. Do, Khue M. Pham, Vinh H. Vu, Linh V. Pham, Lien T. H. Nguyen, Hoang C. Nguyen, Tuan V. Tran, Trung H. Nguyen, Anh T. Nguyen, Hoan V. Nguyen, Phuoc B. Nguyen, Hoai T. T. Nguyen, Thu T. M. Pham, Thuy T. Le, Cuong Q. Tran, Kien T. Nguyen, Han T. Vo, Tuyen Van Duong

**Affiliations:** 1 Department of Psychiatry, Military Hospital 103, Vietnam Military Medical University, Hanoi, 121-08 Vietnam; 2 Centre for Ageing Research & Education, Duke-NUS Medical School, Singapore, 169857 Singapore; 3 Faculty of Public Health, Phenikaa University, Hanoi, 121-16 Vietnam; 4 Institute for Community Health Research, University of Medicine and Pharmacy, Hue University, Hue, 491-20 Vietnam; 5 Director Office, Thai Nguyen National Hospital, Thai Nguyen City, 241-24 Vietnam; 6 Training and Direction of Healthcare Activity Center, Thai Nguyen National Hospital, Thai Nguyen City, 241-24 Vietnam; 7 Biochemistry Department, Thai Nguyen National Hospital, Thai Nguyen City, 241-24 Vietnam; 8 Department of Orthopedics, Can Tho University of Medicine and Pharmacy, Can Tho, 941-17 Vietnam; 9 Director Office, Can Tho University of Medicine and Pharmacy Hospital, Can Tho, 941-17 Vietnam; 10 Department of Infectious Diseases, Vietnam Military Medical University, Hanoi, 121-08 Vietnam; 11 Department of Military Science, Vietnam Military Medical University, Hanoi, 121-08 Vietnam; 12 Faculty of Public Health, Hai Phong University of Medicine and Pharmacy, Hai Phong, 042-12 Vietnam; 13 Infectious and Tropical Diseases Department, Viet Tiep Hospital, Hai Phong, 047-08 Vietnam; 14 Department of Pulmonary & Cardiovascular Diseases, Hai Phong University of Medicine and Pharmacy Hospital, Hai Phong, 042-12 Vietnam; 15 President Office, Thai Nguyen University of Medicine and Pharmacy, Thai Nguyen City, 241-17 Vietnam; 16 Neurology Department, Thai Nguyen University of Medicine and Pharmacy, Thai Nguyen City, 241-17 Vietnam; 17 Director Office, Gang Thep Hospital, Thai Nguyen, 241-34 Vietnam; 18 Director Office, Hospital for Tropical Diseases, Hai Duong, 031-17 Vietnam; 19 Infectious and Tropical Diseases Department, Viet Tiep Hospital, Hai Phong, 047-08 Vietnam; 20 Department of Infectious Diseases, Hai Phong University of Medicine and Pharmacy, Hai Phong, 042-12 Vietnam; 21 Director Office, Kien An Hospital, Hai Phong, 046-09 Vietnam; 22 Training and Direction of Healthcare Activity Center, Kien An Hospital, Hai Phong, 046-09 Vietnam; 23 School of Public Health, College of Public Health, Taipei Medical University, Taipei, 110-31 Taiwan; 24 Da Nang University of Medical Technology and Pharmacy, Da Nang, 502-06 Vietnam; 25 Faculty of Medical Laboratory Science, Da Nang University of Medical Technology and Pharmacy, Da Nang, 502-06 Vietnam; 26 Faculty of Health Sciences, University of Cuu Long, Vinh Long, 852-16 Vietnam; 27 Department of Health Promotion, Faculty of Social and Behavioral Sciences, Hanoi University of Public Health, Hanoi, 119-10 Vietnam; 28 Department of Psychiatry, Hue University of Medicine and Pharmacy, Hue University, Hue, 491-20 Vietnam; 29 International Master/Ph.D. Program in Medicine, College of Medicine, Taipei Medical University, Taipei, 110-31 Taiwan; 30 School of Nutrition and Health Sciences, Taipei Medical University, Taipei, 110-31 Taiwan

**Keywords:** COVID-19 survivors, Health-related quality of life, Fear, Post-traumatic stress symptoms, Latent profile analysis

## Abstract

Survivors of COVID-19 are susceptible to diminished health-related quality of life (HRQoL) and adverse psychological health, which may be exacerbated by their experiences of fear and the impact of the pandemic itself. This study aims to identify distinct fear and impact patterns related to the COVID-19 pandemic among survivors through latent profile analysis (LPA) and examine the associations of fear and impact patterns with post-traumatic stress symptoms (PTSS) and HRQoL. A total of 5,890 Vietnamese COVID-19 survivors completed the COVID-19 Impact Battery– Disability Scale (CIB-D), the Fear of COVID-19 Scale (FCoV-19 S), the Impact of Event Scale-Revised for PTSS, and the 36-Item Short Form Survey (SF-36) for HRQoL. Four distinct groups of fear and impact were identified: “Fearful and highly impacted” (26.8%), “moderately impacted yet not fearful” (22.9%), “less impacted and less fearful” (18.6%), and “mildly impacted and neutral” (31.7%). Survivors who were “less impacted and less fearful” exhibited significantly higher HRQoL scores (regression coefficient, B: 10.9; 95% confidence interval (CI): 10.0 − 11.7), both in terms of physical (B: 12.0; 95%CI: 11.1 − 12.9) and mental health (B: 19.4; 95%CI: 9.6 − 11.1), and lower PTSS levels (B: -24.5; 95%CI: -25.8 − -23.3) compared to those who were “highly impacted and fearful”. It is imperative to acknowledge the intricate association between fear, impact, and mental health to comprehensively address the diverse needs of this distinct population post-COVID-19. These findings provide insights for designing interventions and support mechanisms for COVID-19 survivors.

**Supplementary Information:**

The online version contains supplementary material available at 10.1007/s44197-024-00333-2.

## Introduction

Since 2020, the COVID-19 pandemic has virtually spread to every country and region. While the pandemic’s toll on public health and economies has been staggering, its collateral consequences have manifested in profound psychological and health challenges, especially for COVID-19 survivors (defined as those who received a confirmed positive SARS-CoV-2 test and had recovered from infection) [1–3]. Several studies have reported the health problems among COVID-19 survivors after their infection, including cognitive function decline, fatigue, insomnia, and serious mental health issues such as depressive and anxiety symptoms [4–8]. Among these problems, post-traumatic stress disorder (PTSD) is also frequently reported [5, 9, 10]. PTSD symptoms were observed among survivors of previous epidemics such as SARS and MERS up to six months after discharge from the hospital [11]. Recent studies also showed that PTSD prevailed among 6 to 18% of COVID-19 survivors up to 3 months after their recovery [12–15]. The long-term impact of COVID-19 is also expected to have a spillover effect on COVID-19 survivors’ quality of life (QoL), influencing their future well-being and lifestyle [8, 16, 17]. Previous reports have indicated that post-COVID-19 patients’ health-related quality of life (HRQoL) was impaired from two months to one year after recovery [18, 19]. Even though the prevalence of poor QoL or PTSD among COVID-19 survivors was profound, the mechanism and attributes through which COVID-19 pandemic may shape the mental health outcomes of COVID-19 survivors remains underexplored.

Fear and the negative impact of COVID-19 are considered among the most critical aspects of COVID-19 that impacting population psychological well-being during the pandemic [10, 20–22]. Recent research has delved into the multifaceted relationship between various types of fear and the impact of the COVID-19 pandemic on individuals’ mental well-being. Fear and the negative impact of COVID-19 have been linked to worsened HRQoL in the general population [23–26], while fear of COVID-19 has also been identified as a risk factor for PTSD symptoms, hindering preventive health behaviors [27–30]. However, the literature on this subject has largely neglected the unique challenges faced by COVID-19 survivors themselves. COVID-19 survivors, due to their firsthand experience with the virus, face a more complex array of fears and impacts compared to the general population. The combination of physical illness, long-term health effects, and psychosocial stressors like stigmatization and isolation often places them at an increased risk for PTSS, diminished QoL, and other psychological distress [31–33]. Moreover, COVID-19 survivors may be disproportionately affected by prolonged symptoms, commonly known as “long COVID.” According to studies, long COVID symptoms, including persistent fatigue, respiratory problems, and cognitive difficulties, are presented among approximately 10–30% of those who recover from the initial infection, further exacerbating psychological distress and lower QoL scores [34, 35]. For these individuals, the impact of COVID-19 extends beyond the acute phase of illness, translating into long-term physical and emotional burdens.

In addition to the physical and psychological impacts, COVID-19 survivors often contend with societal stigma. Stigmatization associated with COVID-19 infection has been reported globally, with survivors facing discrimination, social rejection, and blame for the spread of the virus. This stigmatization can lead to prolonged social isolation, further compounding feelings of fear and anxiety and contributing to an increased risk of PTSD, depression, and anxiety [36–39]. The psychological burden of COVID-19 survivors is therefore multifaceted, encompassing the direct impact of the disease, the long-term health consequences, and the social implications of being a COVID-19 survivor. Given this context, there might be an intricate interplay between different dimensions of fear and impact of COVID-19 which can negatively influence psychological well-being and overall QoL of COVID-19 survivors. Therefore, it is crucial to investigate the multifaceted types of experience in fear and impact of COVID-19, and its association with psychological outcomes specific to COVID-19 survivors. By identifying distinct patterns of fear and impact within this population, the findings may offer critical insights into the differential pathways through which COVID-19 could link to poorer psychological well-being and quality of life and inform post-pandemic healthcare strategies for COVID-19 survivors.

While the fear and impact constructs are distinct, it is plausible that they are interconnected within the context of the pandemic. For example, fear can amplify the impact on individuals’ daily functioning, and conversely, the real-world impact of the pandemic can reinforce feelings of fear. Given the interrelationship between fear and impact of COVID-19, we hypothesize that these two constructs may jointly contribute to the psychological outcomes observed in COVID-19 survivors. Therefore, we propose to use latent class analysis (LCA) on both scales to measure various levels of COVID-19 fear and impact among COVID-19 survivors. The LCA is a statistical method for identifying unobserved subgroups within a population based on individual’s responses to a set of categorical observed indicators [40], and we propose to use latent profile analysis (a variant of LCA using continuous indicators), which was used in previous studies to assess the multilayer and multidimensional characteristics of individual’s health and social lives [41]. The LCA method does not assume that fear and impact are identical; rather, it uncovers how these constructs co-occur and manifest in different subgroups within the population. This study aims to identify (a) distinct types of fear and impact of the COVID-19 pandemic experienced by COVID-19 survivors and (b) the association of types of COVID-19 fear and impact with their post-traumatic stress symptoms and health-related quality of life (HRQoL).

## Research Methods

### Study Design and Sample

We utilized data from a cross-sectional online survey conducted among COVID-19 survivors in Vietnam from December 2021 to October 2022. A convenience sample of 11,626 COVID-19 survivors aged 18 years or older was identified at 17 healthcare settings national-wide, including seven hospitals and one health center in the North, two health centers and one isolation facility in the Center, and five hospitals and one health center in the South (Further details in Supplement Table 1). The COVID-19 survivors were contacted by the research team to give consent to participate in the study concerning COVID-19’s impact. Once verbal consent was obtained, participants received a survey web link via phone message or by scanning the quick response (QR) code. An additional online written consent form was collected before the survey commenced. Participants were encouraged to complete all the questionnaires, which took up to 20 min. The survey questionnaire was constructed and administered using Microsoft Forms. A total of 5,890 participants provided consent and completed the online survey, thereby being included in the final analysis.

**Table 1:** Model fit indices for latent groups

	2 Class	3 Class	4 Class	5 Class	6 Class
Log likelihood	-66269.8	-58654.7	-54886.0	-53703.2	-53502.6
AIC	132653.6	117481.4	110002.3	107694.6	106398.2
BIC	133034.4	118056.0	110770.7	108656.7	107356.2
Smallest class (%)	43.6%	27.5%	18.6%	8.4%	3.2%
Entropy	0.98	0.96	0.95	0.95	0.93
*p*(LMR)	0.13	0.19	0.20	0.36	0.40

*Note* AIC: Akaike Information criteria. BIC: Bayern information criteria. LMR: Lo-Mendell Rubin

### Measurements and Assessments

We used the COVID-19 Impact Battery– Disability Scale (CIB-D) [42] and the Fear of COVID-19 Scale (FCoV-19 S) [43] to assess latent groups of COVID-19 impact and fear.

#### COVID-19 Impact

COVID-19 impact was measured by the COVID-19 Impact Battery– Disability Scale (CIB-D) [42]. The CIB-D scale comprises seven items adapted from the World Health Organization Disability Assessment 12-item scale (WHODAS 2.0) and was used to assess difficulties in daily life functioning due to the COVID–19 pandemic. Participants were prompted to rate difficulties that occurred “due to the COVID–19 outbreak” rather than those that arose “due to health conditions.” Respondents scored 7 items (e.g., “How much have you been emotionally affected by the COVID-19 outbreak” or “How much difficulties have you found in taking care of household responsibilities”) using a Likert scale ranging from “None” (scored as 0), “Mild -moderate” (scored as 1), and “Severe - extreme” (scored as 2). The items were specifically designed to evaluate the impact of COVID–19 on household activities, day-to-day work, community activities, emotional health, concentration, getting along with new people, and maintaining friendships. The overall score ranges from 0 to 21, with a higher score indicating a greater disability level due to the impact of COVID-19 (Cronbach’s α = 0.92).

#### COVID-19 Fear

COVID-19 fear was measured by the Fear of COVID-19 Scales (FCoV-19 S) [43]. The FCoV-19 S comprise seven items prompting participants to rate their perceptions and attitude towards COVID-19, and how the pandemic impacts them physically and mentally (e.g., “I am most afraid of COVID-19” or “I feel uncomfortable to think about COVID-19”). Each question requires a response on a 5-point Likert scale from 1 (“Strongly disagree”) to 5 (“Strongly agree”). The sum of seven items ranges from 7 to 35, with a higher score indicating a greater fear and discomfort of COVID-19 (Cronbach’s α = 0.85). This scale has been used and validated in Vietnam [44–46].

#### Post-Traumatic Stress Symptoms

Post-traumatic stress symptoms (PTSS) were measured using the Impact of Event Scale-Revised (IES-R) [47]. The IES-R consists of 22 items that measure how much psychological distress an individual would experience after exposure to a crisis (e.g. “Any reminder brought back feelings about it” or “I had trouble staying asleep). Items are rated on a 5-point scale ranging from “Not at all” (scored as 0); “A little bit” (scored as 1); “Moderately” (scored as 2); “Quite a bit” (scored as 3); and “Extremely” (scored as 4). We created the summed variable of PTSS which ranges from 0 to 88 (Cronbach’s α = 0.83), with a higher score indicating greater post-traumatic stress symptoms.

#### Health-Related Quality of Life

We employed the 36-item Short Form Survey (SF-36) developed by RAND Corporation (RAND-36) to assess health-related quality of life [48, 49], which has been validated in Vietnamese [50]. The RAND-36 comprises of 36 items grouped into eight dimensions (10 items of physical functioning, 2 items of social functioning, 4 items of role limitation in physical problems, 3 items in role limitations in emotional problems, 5 items of mental health, 4 items of vitality, 2 items of pain, 5 items of general health perception, and 1 item of health change). For each dimension, item scores were coded, summed, and transformed on to a scale from “Worst health” (scored as 0) to “Best health” (scored as 100) (Cronbach’s α = 0.80). From item score, two sub-component scores were calculated: the Physical Component Summary (PCS) score (providing overall view score of physical health) and the Mental Component Summary (MCS) score (providing an overall view score pf mental health).

#### Covariates

We accounted for several covariates in our analysis. These include age, gender (1 = Male, 0 = Female), marital status (1 = Never married; 2 = Married; 3 = Widowed/divorced/separated); education level (1 = Illiterate/Elementary; 2 = Junior/ Senior high school; 3 = Vocational/Colleague; 4 = University or above); employment status (1 = Employed; 2 = Unemployed); self-reported income level (1 = low; 2 = middle; 3 = high); comorbidity/ any chronic conditions other than COVID-19 (0 = No; 1 = Yes). We also asked participants to report on their COVID-19 infection: number of times infected with SARS-CoV-2 (1 = Once; 2 = More than once); number of COVID-19 vaccine doses at time of infection (1 = None; 2 = one dose; 3 = more than one dose); COVID-19 severity level (1 = Mild symptoms; 2 = Moderate symptom; 3 = Severe to extremely severe); number of family members infected with SARS-CoV-2; and number of days of hospitalization for COVID-19 treatment. We also included the psychological resilience score of participants, measured by the Vietnamese version [51] of the Brief Resilience Scale (BRS) (final score ranging from 5 to 30, with higher scores indicating higher levels of resilience) [52].

### Data Analysis

First, to derive the types of COVID-19 fear and impact, we fit multiple LPA models with a different number of classes, incorporating 7 items from CIB-D scale and 7 items from the FCoV-19 S scale. Second, we compare models based on the information criteria (Akaike Information Criterion– AIC and Bayesian information criterion– BIC), accuracy (Entropy), parsimony (Lo-Mendell Rubin test), and interpretability (Smallest class (%)) to determine the optimal model. The lower the value of the information criteria (AIC/BIC), the better the fit of the model to the data. A higher entropy indicates a more precise classification. The Lo Mendell Rubin (LMR) test checks whether the model with the k class fits the data better than the model with the k-1 class. Third, we assigned each respondent to a specific group based on the highest posterior probability. Last, we estimated multivariable regression models that examine the association between types of COVID-19 fear and impact and COVID-19 survivors’ PTSS and HRQoL, controlled for all covariates. The descriptive and regression analysis were performed on Stata 15.0 (Stata Corp, College Station, TX, USA), and the LPA models were conducted on M*plus* 8.0 software.

## Results

Descriptive statistics of the 5,890 COVID-19 survivors are presented in Supplement Table 2. The mean of PTSS score was 28.2 ± 18.8 (mean ± standard deviation). The mean of HRQoL was 64.1 ± 12.0, the average score in physical health subdomain score (63.3 ± 13.7) was higher than the average score in mental health subdomain (59.9 ± 10.3). The average COVID-19 impact score of the sample was 17.8 ± 5.3 and the average COVID-19 fear score was 20.4 ± 5.0). The median age of COVID-19 survivors was 31 years, 59% of them were females, 58% were married. 52% have a university or above level of education, about 80% were employed, and 82% reported a middle-income level. The mean resilience score was 18.0 ± 1.9. At the time of infection, 71% of COVID-19 survivors did not have any other chronic disease, 92% received more than one dose of a COVID-19 vaccine, and about 36% experienced moderate to extremely serious symptoms due to their infection. The median of days the participants were hospitalized due to COVID-19 infection was 6.0 days. About 3% of survivors were infected with SARS-CoV-2 more than once, and the median number of same-household-members who got infected with SARS-CoV-2 was 3.0 persons.

**Table 2:** Distribution of health-related quality-of-life score, post-traumatic stress symptoms score, and covariates stratified by latent groups (*n* = 5890)

Factors	Group 1 Highly impacted and fearful (*n* = 1576, 26.8%)	Group 2 Moderately impacted yet least fearful (*n* = 1351, 22.9%)	Group 3 Less impacted and less fearful (*n* = 1094, 18.6%)	Group 4 Mildly impacted and neutral (*n* = 1869, 31.7%)	*p*-value
	*N* (%) or Mean ± SD				
Health related quality of life					
Overall score	59.5 ± 10.5	66.9 ± 11.4	72.5 ± 12.6	61.1 ± 9.9	< 0.001^a^
Physical summary score	57.8 ± 12.6	66.4 ± 13.1	72.3 ± 13.9	60.5 ± 11.6	< 0.001^a^
Mental summary score	56.1 ± 8.4	62.1 ± 9.9	68.0 ± 12.1	56.8 ± 7.4	< 0.001^a^
Post-traumatic stress symptoms score	37.5 ± 17.9	21.8 ± 16.5	11.9 ± 14.2	34.4 ± 15.2	< 0.001^a^
COVID-19 impact score	20.7 ± 4.0	18.3 ± 3.6	9.0 ± 2.4	20.0 ± 2.4	< 0.001^a^
COVID-19 fear score	25.9 ± 3.1	15.8 ± 3.4	17.3 ± 5.2	21.0 ± 0.7	< 0.001^a^
Age (Median, Interquartile range)	32.0 (24.0, 45.0)	29.0 (23.0, 39.0)	32.0 (23.0, 39.0)	31.0 (23.0, 40.0)	0.065^b^
Gender					0.473^c^
Male	526 (33.4)	627 (46.4)	496 (45.3)	798 (42.7)	
Female	1050 (66.6)	724 (53.6)	598 (54.7)	1071 (57.3)	
Marital status					< 0.001^c^
Never married	539 (34.2)	646 (47.8)	433 (39.6)	728 (39.0)	
Married	981 (62.2)	684 (50.6)	625 (57.1)	1112 (59.5)	
Widow/divorce/separate	56 (3.6)	21 (1.6)	36 (3.3)	29 (1.6)	
Education levels					0.068^c^
Illiterate/Elementary	44 (2.8)	24 (1.8)	13 (1.2)	80 (4.3)	
Junior/Senior high school	416 (26.4)	231 (17.1)	145 (13.3)	472 (25.3)	
Vocational/Colleague	496 (31.5)	243 (18.0)	231 (21.1)	461 (24.7)	
University or above	620 (39.3)	853 (63.1)	705 (64.4)	856 (45.8)	
Employment status					0.242^c^
Employed	1,285 (81.5)	1,131 (83.7)	921 (84.2)	1,599 (85.6)	
Unemployed	291 (18.5)	220 (16.3)	173 (15.8)	270 (14.4)	
Income level					0.035^c^
Low	268 (17.0)	161 (11.9)	99 (9.0)	239 (12.8)	
Middle	1233 (78.2)	1120 (82.9)	903 (82.5)	1566 (83.8)	
High	75 (4.8)	70 (5.2)	92 (8.4)	64 (3.4)	
Comorbidity (other than COVID-19)					0.442^c^
No	985 (62.5)	985 (72.9)	859 (78.5)	1348 (72.1)	
Yes	591 (37.5)	366 (27.1)	235 (21.5)	521 (27.9)	
Psychological resilience score	18.1 (1.7)	18.1 (2.1)	17.9 (2.4)	17.9 (1.3)	0.019^c^
Number of times infected by SARS-CoV-2					0.854^c^
Once	1548 (98.2)	1301 (96.3)	1070 (97.8)	1823 (97.5)	
More than once	28 (1.8)	50 (3.7)	24 (2.2)	46 (2.5)	
Number of COVID-19 vaccine doses at time of infection					0.078^c^
None	99 (6.3)	36 (2.7)	63 (5.8)	57 (3.0)	
One dose	103 (6.5)	38 (2.8)	47 (4.3)	56 (3.0)	
More than one	1374 (87.2)	1277 (94.5)	984 (89.9)	1756 (94.0)	
COVID-19 severity level					0.031^c^
Mild	883 (56.0)	924 (68.4)	816 (74.6)	1141 (61.0)	
Moderate	648 (41.1)	414 (30.6)	267 (24.4)	713 (38.1)	
Severe to extreme	45 (2.9)	13 (1.0)	11 (1.0)	15 (0.8)	
Number of family member infected with SARS-CoV-2 (Median, Interquartile range)	4.0 (1.0, 5.0)	3.0 (1.0, 4.0)	3.0 (1.0, 5.0)	3.0 (1.0, 6.0)	0.060^b^
Number of days of hospitalization for COVID-19 treatment (Median, Interquartile range)	6.0 (2.0, 10.0)	6.0 (1.0,11.0)	6.0 (1.0, 9.0)	6.0 (1.0, 8.0)	0.350^b^

*Note*. *p*-value was calculated by ^a^ANOVA, ^b^Mann-Whitney test, and ^c^Chi-square test

COVID-19: Corona virus disease of 2019; SARS-CoV-2: Severe acute respiratory syndrome coronavirus 2

Table 1 reports the model fit indices of LPA models from two to six classes. Across all models, the entropy indices are all above 0.90 and the LMR test is insignificant. Although models with lower AIC and BIC values fit the data better, the six-class model has a negligible number in the smallest class (less than 5%), we thus exclude the six-class option [53–55]. The reduction in AIC and BIC levels off after the five-class model, however, the four-class model is more parsimonious and easier to interpret than the five-class model. Therefore, we chose the four-class model.

**Fig. 1:**
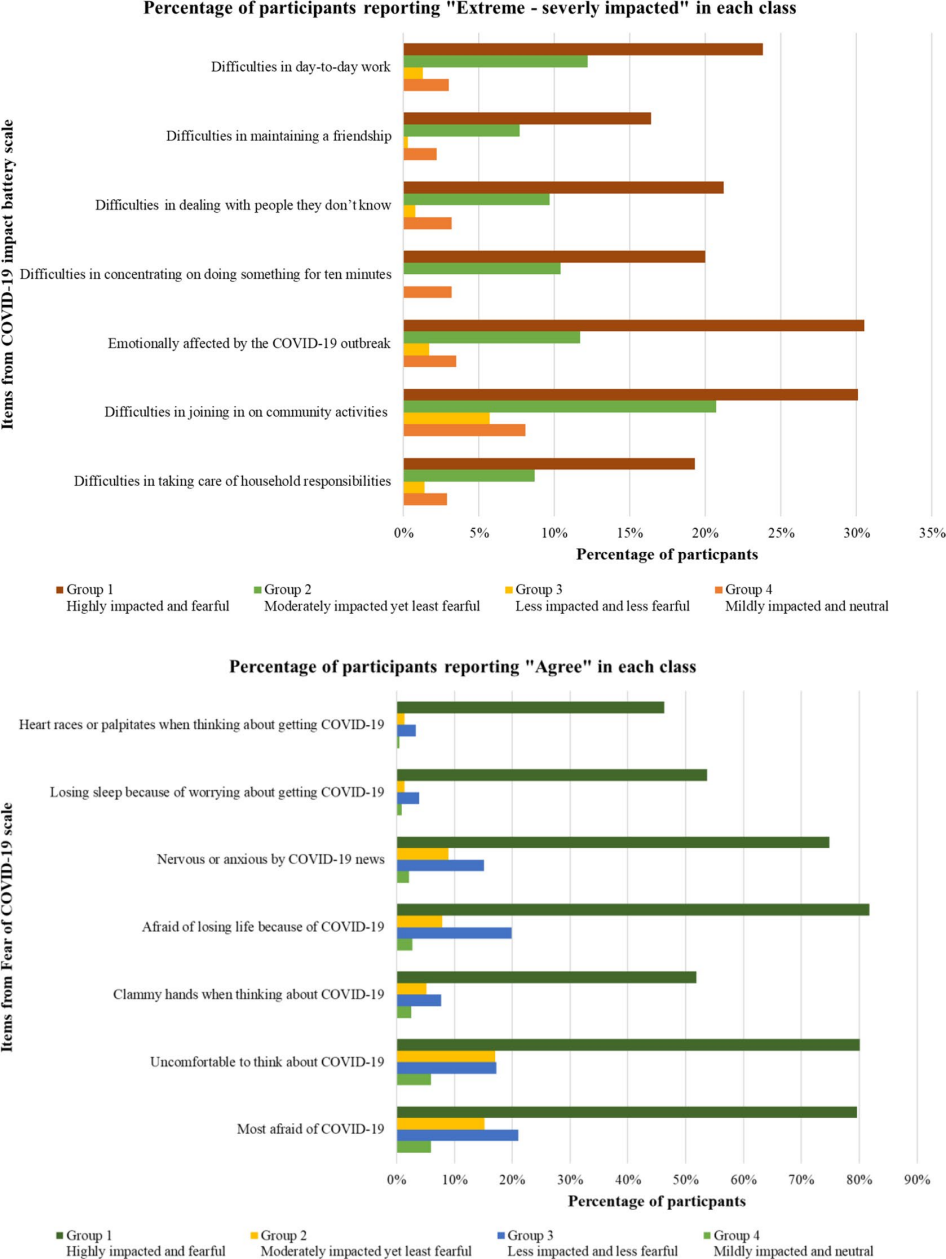
Distribution of COVID-19 fear and impact among groups. Note. Figure only displays “Severe-extremely severe” or “Agree” categories percentage. More details can be found in Supplement Table 2

Figure 1 and Supplement Table 3 display the profile of the four-group model. The first group, comprising 27% of participants, represents survivors with the highest COVID-19 impact and COVID-19 fear scores and is labeled “Fearful and highly impacted”. The second group represents COVID-19 survivors, constituting 23% of participants, were the second-most impacted by COVID-19, yet the least fearful of the pandemic in the sample size– thus termed as “moderately impacted yet not fearful” group. The third group, comprising 19% of participants, includes those who were the least impacted by COVID-19 and less fearful of the disease and/or the pandemic– labelled as “less impacted and less fearful”. The fourth and largest group (31.7% of participants) represents COVID-19 survivors who were mildly impacted by COVID-19, and the second most fearful of COVID-19. We label this group as “mildly impacted and neutral”.

**Table 3:** Association between sociodemographic characteristics, COVID-19 infection and latent groups of fear and impact of COVID-19 based on multinomial logistic regression (*n* = 5890)

Factors	Group 2	Group 3	Group 4
	B (95% CI)		
Age	-0.001 (-0.01 − 0.01)	-0.004 (-0.01 − 0.004)	-0.003 (-0.01 − 0.002)
Gender			
Male	Ref.	Ref.	Ref.
Female	-0.4 (-0.6 − -0.3)^***^	-0.4 (-0.6 − -0.2)^***^	-0.3 (-0.5 − -0.2)^***^
Marital status			
Never married	Ref.	Ref.	Ref.
Married	-0.1 (-0.3 − 0.1)	0.3 (0.1 − 0.6)^**^	-0.04 (-0.2 − 0.1)
Widow/divorce/separate	-0.5 (-1.1 − 0.03)	0.7 (0.2 − 1.2)^**^	-0.5 (-1.0 − -0.02)^*^
Education levels			
Illiterate/Elementary	Ref.	Ref.	Ref.
Junior/ Senior high school	-0.1 (-0.7 − 0.4)	0.1 (-0.5 − 0.8)	-0.6 (-1.0 − -0.2)^**^
Vocational/Colleague	-0.4 (-0.9 − 0.1)	0.4 (-0.3 − 1.0)	-0.9 (-1.4 − -0.5)^***^
University or above	0.4 (-0.2 − 0.9)	1.2 (0.5 − 1.8)^***^	-0.6 (-1.1 − -0.2)^**^
Employment status			
Employed	Ref.	Ref.	Ref.
Unemployed	-0.2 (-0.4 − 0.04)	-0.01 (-0.2 − 0.2)	-0.2 (-0.4 − -0.02)^*^
Income level			
Low	Ref.	Ref.	Ref.
Middle	0.3 (0.1 − 0.5)^**^	0.5 (0.2 − 0.8)^***^	0.3 (0.1 − 0.5)^**^
High	0.1 (-0.3 − 0.5)	0.7 (0.3 − 1.1)^***^	-0.1 (-0.5 − 0.2)
Comorbidities (other than COVID-19)			
No	Ref.	Ref.	Ref.
Yes	-0.2 (-0.4 − -0.1)^**^	-0.5 (-0.7 − -0.3)^***^	-0.3 (-0.5 − -0.2)^***^
Psychological resilience score	0.001 (-0.04 − 0.04)	-0.03 (-0.1 − 0.01)	-0.04 (-0.1 − -0.01)^*^
Number of times infected by SARS-CoV-2			
Once	Ref.	Ref.	Ref.
More than once	0.8 (0.3 − 1.3)^***^	0.3 (-0.3 − 0.9)	0.3 (-0.1 − 0.8)
Number of COVID-19 vaccine doses at time of infection			
None	Ref.	Ref.	Ref.
One dose	-0.03 (-0.6 − 0.5)	-0.3 (-0.8 − 0.1)	-0.1 (-0.6 − 0.4)
More than one	0.8 (0.4 − 1.2)^***^	-0.02 (-0.4 − 0.3)	0.8 (0.4 − 1.1)^***^
COVID-19 severity level			
Mild	Ref.	Ref.	Ref.
Moderate	-0.3 (-0.5 − -0.1)^***^	-0.7 (-0.9 − -0.5)^***^	-0.1 (-0.2 − 0.1)
Severe to extreme	-0.9 (-1.6 − -0.3)^**^	-1.1 (-1.8 − -0.4)^**^	-1.1 (-1.7 − -0.5)^***^
Number of family member infected with SARS-CoV-2	-0.06 (-0.1 − -0.01)^**^	-0.01 (-0.05 − 0.03)	0.02 (-0.01 − 0.04)
Number of days of hospitalization for COVID-19 treatment	0.002 (-0.01 − 0.01)	0.01 (-0.003 − 0.02)	0.01 (0.003 − 0.02)^*^

*Note*. ^*^p-value < 0.05, ^**^p-value < 0.01, ^***^p-value < 0.001. Group 2, 3, and 4 were compared with Group 1– Highly impacted and fearful

B: unstandardized regression coefficient; CI: confidence interval; COVID-19: Corona virus disease of 2019; SARS-CoV-2: Severe acute respiratory syndrome coronavirus 2

Across domains of both scales, survivors in the first group “fearful and highly impacted” reported the highest percentage of severe to extremely severe difficulties across several aspects of life amidst the COVID-19 pandemic, about one-third of them had challenges participating in community activities and experienced emotional impacts by the pandemic. They also experienced a lot of nervousness and anxiety due to COVID-19, as 80% of participants in this group felt uncomfortable or afraid of losing their lives. Survivors in “moderately impacted yet not fearful” group showed a lower score in impact of COVID-19 scale than participants in the first group, however, they had the least fearful score across all domains. Notably, a high percentage of this group did not experience any negative physical challenges (such as nervousness, sleep disturbance) nor were afraid of losing their lives due to the pandemic. Survivors in the third group - “less impacted and less fearful” had the lowest impact score by COVID-19 among all groups and exhibited a lower level of fear. Despite accounting for the smallest proportion, more than 70% of participants in this group reported no difficulties in performing daily activities after recovering from their SARS-CoV-2 infection. More than 90% of survivors in the fourth group - “mildly impacted and neutral” - experienced mild to moderate levels of difficulties in certain activities due to COVID-19. About 90% of them also reported neutral feelings towards being fear of COVID-19, and they contributed the smallest proportion that being physically impacted while thinking about COVID-19.

Table 2 provides the distribution of HRQoL score, PTSS score, COVID-19 fear and impact score, and covariates stratified by latent groups. There is a significant distinction between survivors who were classified in the “highly impacted and fearful” group or “mildly impacted and neutral” group towards COVID-19 and survivors who were in the “less impacted and less fearful” group or “moderately impacted yet least fearful” group: while the former survivors reported a higher PTSS score and a lower HRQoL score (overall and in subdomains) compared to the latter. There are no statistical differences in the distribution of all covariates among the four groups, except for marital status, income level, psychological resilience score, COVID-19 severity level, and the number of family members infected with COVID-19. Notably, survivors who were in the “highly impacted and fearful” group have the highest percentage of low income, the highest proportion of chronic diseases, and the highest percentage of having severe to extremely severe COVID-19 symptoms. Participants in this group also had the highest proportion of being unvaccinated before their infection among other survivor groups. About 13% of survivors in the “mildly impacted and neutral” group reported in the low-income group, about 30% of them have chronic conditions, and they also had the lowest psychological resilience score, and the highest number of family members also infected with SARS-CoV-2.

Multinominal logistic regression in Table 3 indicates that gender, income level, education level, marital status, comorbidities other than COVID-19, and COVID-19 severity level were significantly associated with membership in the “highly impacted and fearful of COVID-19” group. Female participants with middle income level (compared to those with lower income level), comorbidities, less than one dose of COVID-19 vaccine at the time of infection, and suffered moderate, severe to extremely severe COVID-19 symptoms during infection were more likely to be a member in the first group “highly impacted and fearful of COVID-19”. In addition, individuals with a high-income level or have ever been married were more likely to be a member in the third group “less impacted and less fearful” than being in the first group. On the other hand, those with a higher education level, middle income, had a higher psychological resilience score or a higher number of hospitalized days were more likely to be a member of the fourth group “mildly impacted and neutral” than in the first group.

Table 4 reports findings from multivariable linear regressions assessing the association between groups of COVID-19 fear and impact with post-traumatic stress symptoms score (Model 1) and HRQoL score (overall and subdomains) (Model 2–4, respectively). All models are controlled for covariates. The regression analysis confirms the descriptive analysis, COVID-19 survivors in the “less impacted and less fearful” group had the lowest PTSS score and the highest HRQoL scores among all other subgroups, and survivors in the “highly impacted and fearful” group scored the reverse. Compared to survivors in the first group “highly impacted and fearful”, survivors in the second group “moderately impacted yet least fearful” (regression coefficient B: 5.9; 95% confidence interval, 95%CI: 15.2 − 6.1 for overall score); the third group “mildly impacted and fearful” (B: 10.9; 95%CI: 10.0 − 11.7), and the fourth group “less impacted and less fearful” (B: 0.9; 95%CI: 0.2 − 1.6) had significantly higher scores of HRQoL, overall and across physical and mental health domains. Survivors in the second group (B: -15.8; 95%CI: -16.9 − -14.6), third group (B: -24.5; 95%CI: -25.8 − -23.3), and fourth group (B: -3.3, 95%CI: -4.3 − -2.2) had significantly lower PTSS score than those in the first group.

**Table 4:** Regression analysis of groups of COVID-19 fear and impact and health-related quality of life score and post-traumatic stress symptoms score (*n* = 5890)

Factors	Model 1: Post-traumatic stress symptoms	Health-related quality of life		
		Model 2: Overall	Model 3: Physical health	Model 4: Mental health
	B (95%CI)			
Group 1 Highly impacted and fearful	Ref.	Ref.	Ref.	Ref.
Group 2 Moderately impacted yet least fearful	-15.8 (-16.9– -14.6)^***^	5.9 (5.2–6.1)^***^	6.8 (5.9–7.7)^***^	4.9 (4.3–5.7)^***^
Group 3 Less impacted and less fearful	-24.5 (-25.8– -23.3)^***^	10.9 (10.0–11.7)^***^	12.0 (11.1–12.9)^***^	19.4 (9.6–11.1)^***^
Group 4 Mildly impacted and neutral	-3.3 (-4.3– -2.2)^***^	0.9 (0.2–1.6)^*^	1.9 (1.1–2.7)^***^	0.4 (-0.2–1.0)
Adjusted R-squared	31.0	27.4	27.5	24.7

Note. All covariates are controlled for but not shown for the parsimonious presentation. ^*^p-value < 0.05, ^**^p-value < 0.01, ^***^p-value < 0.001. B: unstandardized regression coefficient; CI: confidence interval

All models included four groups of COVID-19 fear and impact and all covariates as independent variables. Model 1 has post-traumatic stress symptom score as dependent variable. Model 2 has the overall score of health-related quality of life as dependent variable. Model 3 and 4 has health-related quality of life in physical health and social health domain as dependent variables, respectively

## Discussion

To our knowledge, this is one of the few studies to explore the heterogeneity in fear and impact of COVID-19 among a large sample of COVID-19 survivors. We identified four distinct latent groups based on the fear and impact of COVID-19. Among these four, participants of three groups experienced similar impact of COVID-19 yet varied in the level of fear towards COVID-19, while participants of one group experienced the least impact and the least fear. We also found that the magnitude of associations between each group and a range of psychological distress and quality of life measures differed significantly. Specifically, individuals in the “highly impacted and fearful” group had significantly lower HRQoL scores in both physical and mental health, along with higher PTSS scores. On the other hand, individuals of the “moderately impacted yet least fearful” group had relatively better HRQoL scores and a lower PTSS score than those in the “mildly impacted and neutral” group.

The four-group model we reported in this study differs slightly from those in previous studies identifying a three-group model of fear of COVID-19 in the general population [56–58]. This discrepancy can be attributed to variations in included indicators and scales in each model. However, current literature also found a similar distinction between high, moderate, and low levels of fear among participants during COVID-19 [56–58]. In our study, within 80% of COVID-19 survivors who were impacted by the COVID-19 pandemic at different intensities (in this study as group 1, 2, and 4), varying levels of fear towards the virus and the pandemic are evident. While a substantial proportion of survivors in the “highly impacted and fearful” group and “moderately impacted yet not fearful” group experienced mildly to moderately difficulties in performing activities due to COVID-19, around 70% of those in the “moderately impacted yet not fearful” group did not report any physical or mental challenges while being exposed to COVID-19-related information compared to only 2 to 10% of those in the “fearful and highly impacted” group reporting in the same categories. Whereas more than 90% of survivors in the “mildly impacted and neutral” group mostly reported a neutral view towards COVID-19. This finding emphasizes the nuanced relationship between fear and impact of COVID-19, indicating that they are not mutually exclusive.

The identification of distinct groups based on fear and impact scores sheds in our study light on the heterogeneous psychological responses to the pandemic, specifically among COVID-19 survivors. We found that Survivors in the “moderately impacted yet not fearful” reported a comparatively lower impact but the least fear, which aligns with studies suggesting that individuals with lower fear levels tend to exhibit better mental health outcomes even in the face of significant stressors. In contrast, survivors in the “less impacted and less fearful” group reported the lowest COVID-19 impact and fear scores, consistent with previous literature highlighting that individuals with minimal exposure to the virus and lower fear levels tend to experience fewer psychological sequelae [59]. Interestingly, the “Mildly impacted and neutral” survivors exhibited a paradoxical response, reporting moderate difficulties but neutrality in fear. This finding corresponds with research suggesting that moderate levels of fear can motivate individuals to adopt protective behaviors [60–62].

Our findings also show that female COVID-19 survivors were more likely to be fearful and impacted by COVID-19 than their male counterparts. This aligns with previous studies reporting on fear among the general population during COVID-19, as women exhibited a higher susceptibility to psychological distress [56, 57, 63]. In addition, being a female COVID-19 survivor had been considered as a predictor for prolonged COVID-19 symptoms and higher PTSD symptom severity [64, 65]. As the disproportionate burden of anxiety and other mental health problems is evidently profound and skewed towards women during the pandemic, our findings further indicated that fear and impact of COVID-19 might be contributing more to this gendered burden. In addition, lower socio-economic status is also a risk factor of being highly impacted and fearful of COVID-19. This finding aligns with existing COVID-19 literature, where financial strains and fear of economic repercussions, which are often tied to job loss or financial instability due to the pandemic, are linked to increased fear and anxiety [65–68]. Furthermore, COVID-19 survivors with comorbidities and more severe COVID-19 symptoms were more likely to be in the “fearful and highly impacted” group. This highlights a “duo effect” of comorbidity, as individuals with comorbidity are not only at higher risk of acquiring COVID-19 infection but also for severe COVID-19 conditions. Previous studies have consistently shown that patients with more severe COVID-19 symptoms face a higher risk of enduring substantial neurological, psychiatric, and psychological morbidity from six months to one year after COVID-19 infection [69–73]. Chan et al. and Luna-Rodriguez et al. both found that psychological problems were more prominent among hospitalized COVID-19 patients [33, 74]. The connection between the severity of COVID-19 infection and psychological distress were explained that the patients were often kept in confinement without family interaction, they also got exposed frequently to negative news about COVID-19 or developed concern about themselves and family health [33, 74, 75]. Overall, our results carry important implications for healthcare services, emphasizing the need to consider the gender-specific and socio-economic factors when identifying and providing healthcare for COVID-19 survivors, who were more vulnerable to complications of the diseases itself.

Next, our study underscores the intricate association between types of fear and impact of COVID-19 with PTSD symptoms, and HRQoL among COVID-19 survivors. Notably, being a survivor in the “fearful and highly impacted” group was associated with significantly higher level of PTSS and lower scores of HRQoL, while being a survivor in the “less impacted and less fearful” group was associated with the reverse. This aligns with existing literature, which has consistently demonstrated that heightened fear of COVID-19 is associated with an increased risk of developing PTSD-like symptoms [76, 77]. These findings further resonate with previous research indicating that elevated fear and perception of threat during pandemics can contribute to long-lasting psychological distress as well as lower quality of life [3, 58, 67, 78–81].

Our study is not without limitations. Firstly, the cross-sectional design of our study does not permit the establishment of causal inference between predictors and outcomes. Therefore, caution consideration is needed when interpreting our results in the general population, as COVID-19 survivors had different pandemic challenges and experiences during infection or after recovery. Secondly, the self-reported online survey method is vulnerable to selection bias and recall bias, since we did not collect the data from participants at point of recovery from SARS-CoV-2 infection. Self-rating scales were used for all scales and measurements, which may influence the accuracy of the results and limit the generalizability of our findings. Thirdly, fear and impact of COVID-19 might not reflect in the total psychological impact and responses of COVID-19 survivors to the pandemic, which may lead to an underestimation of their mental well-being. Fourth, we acknowledged that there might be a multicollinear relationship between fear and impact of COVID-19. However, to mitigate potential confounding, we examined the correlation between all items within the fear and impact of COVID-19 scales during preliminary analyses and did not find a strong correlation between the two scales. We also adjusted for other variables that could confound the relationship between these constructs, such as sociodemographic factors and prior health conditions.

Our study carries several notable strengths. The application of latent profile analysis yielded four distinct groups that shed light on the multifaceted psychological responses within this growing cohort of COVID-19 recovered population. This approach also allows for a clearer understanding of how individuals with varying levels of fear and impact cope with the aftermath of COVID-19, thus reaffirming the potential association of the long-term impacts of COVID-19 on psychological health and QoL. Our findings provide a foundation for tailoring interventions and support for this population, recognizing the need for a holistic understanding of their experiences.

## Conclusion

By identifying four distinct groups based on fear and impact of COVID-19 among COVID-19 survivors, our result provides insights into the heterogeneous psychological responses of survivors to the pandemic. The findings also underscore the significant association between types of impact and fear of COVID-19, on mental health and well-being. These findings are a foundation for tailoring the strategical interventions and support for COVID-19 survivors. Recognizing the complex interplay of fear, impact, and mental health helps addressing the holistic needs of COVID-19 survivors to navigate the recovery from infection.

## Electronic supplementary material

Below is the link to the electronic supplementary material.

Supplementary Material 1

## Data Availability

No datasets were generated or analysed during the current study.

## Abbreviations

AIC: Akaike Information Criterion
BIC: Bayesian information criterion
BRS: Brief Resilience Scale
CIB-D: COVID-19 Impact Battery– Disability Scale
FCoV-19S: Fear of COVID-19 Scale
HRQoL: Health-related Quality of Life
IES-R: Impact of Event Scale-Revised
LCA: Latent class Analysis
LMR: Lo Mendell Rubin
LPA: Latent Profile Analysis
QoL: Quality of Life
QR: Quick Response
PTSD: Post-traumatic stress disorder
PTSS: Post-traumatic stress symptoms
SD: Standard deviation
SF-36: 36-Item Short Form Survey
