# Supplementary material for: Fear and Impact of COVID-19 Among Post-Infected Adults: Types and Associations with Quality of Life and Post-Traumatic Stress Symptoms
Source: J Epidemiol Glob Health. 2024 Dec 2;14(4):1748–63. doi: 10.1007/s44197-024-00333-2 (PMC11652562; doi:10.1007/s44197-024-00333-2)
Supplement: Supplementary file 1 — Supplementary Material 1 [file 44197_2024_333_MOESM1_ESM.docx]

**Article Title:** Fear and Impact of COVID-19 Among Post-Infected Adults: Types and Associations with Quality of Life and Post-Traumatic Stress Symptoms
**DOI:** 10.1007/s44197-024-00333-2

**Supplement Table 1. Distribution of participants recruited across Vietnam.**

| **Region of Vietnam** | **City/ Province** | **Participated COVID-19 survivors** | **Count of COVID-19 infections ^a^** | **Count of COVID-19 deaths ^a^** |
| --- | --- | --- | --- | --- |
| Northern | Hai Duong | 111 | 362,258 | 114 |
|  | Hai Phong | 1,457 | 119,396 | 135 |
|  | Ha Noi | 906 | 1,600,496 | 1,221 |
|  | Thai Nguyen | 976 | 184,288 | 110 |
| Central | Thua Thien Hue | 595 | 46,239 | 173 |
|  | Quang Tri | 287 | 81,112 | 38 |
|  | Da Nang | 428 | 97,573 | 337 |
| Southern | Ho Chi Minh | 625 | 611,040 | 19,984 |
|  | Can Tho | 505 | 76,380 | 950 |
| ^a^ According to latest reports on June 2022 by Vietnam Ministry of Health, <http://covid19.ncsc.gov.vn/dulieu> | | | | |

**Supplement Table 2. Descriptive analysis of all sample sizes.**

| **Factor** | **N (%) or mean ± SD or median (IQR)** |
| --- | --- |
| **N** | 5890 |
| Post-traumatic stress symptoms score | 28.2 **±**18.8 |
| Quality of life |  |
| Overall score | 64.1 **±**12.0 |
| Physical summary score | 63.3 **±**13.7 |
| Mental summary score | 59.9 **±**10.3 |
| COVID-19 impact score | 17.8 **±**5.3 |
| COVID-19 fear score | 20.4 **±**5.0 |
| Age | 31.0 (23.0, 40.0) |
| Gender |  |
| Male | 2447 (41.5) |
| Female | 3443 (58.5) |
| Marital status |  |
| Never married | 2346 (39.8) |
| Married | 3402 (57.8) |
| Widowed/divorce/separate | 142 (2.4) |
| Education levels |  |
| Illiterate/Elementary | 161 (2.7) |
| Junior/ Senior high school | 1264 (21.5) |
| Vocational/Colleague | 1431 (24.3) |
| University or above | 3034 (51.5) |
| Employment status |  |
| Employed | 4936 (83.8) |
| Unemployment | 954 (16.2) |
| Income level |  |
| Low | 767 (13.0) |
| Middle | 4822 (81.9) |
| High | 301 (5.1) |
| Comorbidity (other than COVID-19) |  |
| No | 4177 (70.9) |
| Yes | 1713 (29.1) |
| Psychological resilience score | 18.0 **±**1.9 |
| Number of times infected by SARS-CoV-2 |  |
| Once | 5742 (97.5) |
| More than once | 148 (2.5) |
| Number of COVID-19 vaccine doses at time of infection |  |
| None | 255 (4.3) |
| One dose | 244 (4.1) |
| More than one | 5391 (91.5) |
| COVID-19 severity level |  |
| Mild | 3764 (63.9) |
| Moderate | 2042 (34.7) |
| Severe to extreme | 84 (1.4) |
| Number of family member infected with SARS-CoV-2 | 3.0 (1.0, 4.0) |
| Number of days of hospitalization for COVID-19 treatment | 6.0 (5.0, 7.0) |
| Note. COVID-19: Corona virus disease of 2019; IQR: Interquartile range; SARS-CoV-2: Severe acute respiratory syndrome coronavirus 2; SD: Standard deviation. | |

**Supplement Table 3. Profiles of latent classes by distribution of COVID-19 fear and impact.**

| **Factor** | **Class 1 Highly impacted and fearful** | **Class 2**  **Moderately impacted yet least fearful** | **Class 3**  **Less impacted and less fearful** | **Class 4 Mildly impacted and neutral** | **p-value** |
| --- | --- | --- | --- | --- | --- |
|  | **N (%) or Mean ±SD** | | | | |
| N | 1576 (26.8) | 1351 (22.9) | 1094 (18.6) | 1869 (31.7) |  |
| *COVID-19 impact battery scale* | | | | | |
| COVID-19 impact score | 20.7 **±**4.0 | 18.3 **±**3.6 | 9.0 **±**2.4 | 20.0 **±**2.4 | <0.001^a^ |
| Difficulties in taking care of household responsibilities | | | | | <0.001^b^ |
| None | 109 (6.9) | 160 (11.8) | 833 (76.1) | 93 (5.0) |  |
| Mild-moderate | 1163 (73.8) | 1073 (79.4) | 246 (22.5) | 1721 (92.1) |  |
| Severe – extreme | 304 (19.3) | 118 (8.7) | 15 (1.4) | 55 (2.9) |  |
| Difficulties in joining in on community activities | | | | | <0.001^b^ |
| None | 51 (3.2) | 62 (4.6) | 734 (67.1) | 18 (1.0) |  |
| Mild-moderate | 1051 (66.7) | 1009 (74.7) | 298 (27.2) | 1699 (90.9) |  |
| Severe to extreme | 474 (30.1) | 280 (20.7) | 62 (5.7) | 152 (8.1) |  |
| Emotionally affected by the COVID-19 outbreak | | | | | <0.001^b^ |
| None | 15 (1.0) | 115 (8.5) | 829 (75.8) | 25 (1.3) |  |
| Mild-moderate | 1080 (68.5) | 1078 (79.8) | 246 (22.5) | 1779 (95.2) |  |
| Severe to extreme | 481 (30.5) | 158 (11.7) | 19 (1.7) | 65 (3.5) |  |
| Difficulties in concentrating on doing something for ten minutes | | | | | <0.001^b^ |
| None | 98 (6.2) | 176 (13.0) | 949 (86.7) | 38 (2.0) |  |
| Mild-moderate | 1163 (73.8) | 1035 (76.6) | 145 (13.3) | 1771 (94.8) |  |
| Severe – extreme | 315 (20.0) | 140 (10.4) | 0 (0.0) | 60 (3.2) |  |
| Difficulties in dealing with people they don’t know | | | | | <0.001^b^ |
| None | 84 (5.3) | 133 (9.8) | 950 (86.8) | 22 (1.2) |  |
| Mild-moderate | 1158 (73.5) | 1087 (80.5) | 135 (12.3) | 1787 (95.6) |  |
| Severe – extreme | 334 (21.2) | 131 (9.7) | 9 (0.8) | 60 (3.2) |  |
| Difficulties in maintaining a friendship | | | | | <0.001^b^ |
| None | 204 (12.9) | 207 (15.3) | 1038 (94.9) | 63 (3.4) |  |
| Mild-moderate | 1114 (70.7) | 1040 (77.0) | 53 (4.8) | 1764 (94.4) |  |
| Severe – extreme | 258 (16.4) | 104 (7.7) | 3 (0.3) | 42 (2.2) |  |
| Difficulties in day-to-day work | | | | | <0.001^b^ |
| None | 116 (7.4) | 73 (5.4) | 943 (86.2) | 35 (1.9) |  |
| Mild-moderate | 1085 (68.8) | 1113 (82.4) | 137 (12.5) | 1778 (95.1) |  |
| Severe – extreme | 375 (23.8) | 165 (12.2) | 14 (1.3) | 56 (3.0) |  |
| *Fear of COVID-19 scale* | | | | | |
| COVID-19 fear score | 25.9 **±**3.1 | 15.8 **±**3.4 | 17.3 **±**5.2 | 21.0 **±**0.7 | <0.001^a^ |
| Most afraid of COVID-19 | | | | | <0.001^b^ |
| Disagree | 73 (4.6) | 645 (47.7) | 380 (34.7) | 78 (4.2) |  |
| Neutral | 249 (15.8) | 501 (37.1) | 483 (44.1) | 1678 (89.8) |  |
| Agree | 1254 (79.6) | 205 (15.2) | 231 (21.1) | 113 (6.0) |  |
| Uncomfortable to think about COVID-19 | | | | | <0.001^b^ |
| Disagree | 29 (1.8) | 563 (41.7) | 373 (34.1) | 45 (2.4) |  |
| Neutral | 284 (18.0) | 557 (41.2) | 532 (48.6) | 1712 (91.6) |  |
| Agree | 1263 (80.1) | 231 (17.1) | 189 (17.3) | 112 (6.0) |  |
| Clammy hands when thinking about COVID-19 | | | | | <0.001^b^ |
| Disagree | 223 (14.1) | 1050 (77.7) | 675 (61.7) | 107 (5.7) |  |
| Neutral | 536 (34.0) | 231 (17.1) | 335 (30.6) | 1716 (91.8) |  |
| Agree | 817 (51.8) | 70 (5.2) | 84 (7.7) | 46 (2.5) |  |
| Afraid of losing life because of COVID-19 | | | | | <0.001^b^ |
| Disagree | 37 (2.3) | 948 (70.2) | 493 (45.1) | 45 (2.4) |  |
| Neutral | 250 (15.9) | 296 (21.9) | 383 (35.0) | 1773 (94.9) |  |
| Agree | 1289 (81.8) | 107 (7.9) | 218 (19.9) | 51 (2.7) |  |
| Nervous or anxious by COVID-19 news | | | | | <0.001^b^ |
| Disagree | 29 (1.8) | 863 (63.9) | 481 (44.0) | 29 (1.6) |  |
| Neutral | 368 (23.4) | 367 (27.2) | 448 (41.0) | 1799 (96.3) |  |
| Agree | 1179 (74.8) | 121 (9.0) | 165 (15.1) | 41 (2.2) |  |
| Losing sleep because of worrying about getting COVID-19 | | | | | <0.001^b^ |
| Disagree | 157 (10.0) | 1172 (86.8) | 691 (63.2) | 19 (1.0) |  |
| Neutral | 573 (36.4) | 160 (11.8) | 360 (32.9) | 1833 (98.1) |  |
| Agree | 846 (53.7) | 19 (1.4) | 43 (3.9) | 17 (0.9) |  |
| Heart races or palpitates when thinking about getting COVID-19 | | | | | <0.001^b^ |
| Disagree | 145 (9.2) | 1179 (87.3) | 681 (62.2) | 29 (1.6) |  |
| Neutral | 701 (44.5) | 153 (11.3) | 377 (34.5) | 1830 (97.9) |  |
| Agree | 730 (46.3) | 19 (1.4) | 36 (3.3) | 10 (0.5) |  |

*Note. P-value was calculated by ^a^ANOVA and ^b^Chi-square test.*

COVID-19: Corona virus disease of 2019; SARS-CoV-2: Severe acute respiratory syndrome coronavirus 2; SD: Standard deviation.
